# Small Bowel Metastasis as a Presentation of Testicular Seminoma

**DOI:** 10.7759/cureus.17962

**Published:** 2021-09-14

**Authors:** Adel Muhanna, Faisal Nimri, Zaid A Almomani, Laith Al Momani, Alisa Likhitsup, Fadi Hamid

**Affiliations:** 1 Internal Medicine, University of Missouri-Kansas City (UMKC) School of Medicine, Kansas City, USA; 2 Internal Medicine, Henry Ford Health System, Detroit, USA; 3 Internal Medicine, Jordan University of Science and Technology, Irbid, JOR; 4 Gastroenterology, University of Missouri-Kansas City (UMKC), Kansas City, USA; 5 Hepatology, University of Missouri-Kansas City (UMKC), Kansas City, USA

**Keywords:** endoscopy, abdominal pain, small bowel metastasis, germ cell tumors, seminoma

## Abstract

Testicular germ cell tumors account for 95% of testicular cancers in men with approximately 71,000 patients being diagnosed with testicular cancer every year. The overall survival of testicular germ cell tumors is approximately 95%. However, the prognosis becomes less favorable when distant metastasis is present. Gastrointestinal (GI) tract metastasis occurs in less than 5% of patients with non-seminomatous tumors, and in less than 1% in patients with pure seminomas. GI metastasis usually involves the colon, esophagus, and stomach with the most common symptoms of GI metastasis being diarrhea, nausea, vomiting, and obstruction. We discuss the case of a 42-year-old male patient with GI manifestations as the first presentation of testicular seminoma with metastasis to the small bowel. Computed tomography of the abdomen and pelvis revealed a small bowel mass, and the diagnosis was confirmed with histopathologic examination of endoscopic biopsy samples. The patient subsequently underwent chemotherapy treatment with close surveillance. Clinicians should maintain a high index of suspicion in the differential diagnosis of abdominal pain in young male patients, especially when associated with symptoms like unexplained weight loss, constitutional symptoms, and testicular pain or swelling. Metastasis to the GI tract from the testis should be promptly diagnosed and managed, as the overall survival rates can significantly decrease with the delay of diagnosis.

## Introduction

Testicular malignancies account for 1% of cancers in men, and most commonly affect young males between 15 and 35 years of age, with germ cell tumors accounting for the majority of testicular cancers [1]. There are approximately 71,000 new cases of testicular cancer per year, and the incidence appears to be rising [2,3]. Testicular cancer most commonly presents as a painless nodule in the testicle, and other less common symptoms such as abdominal pain, gynecomastia, or testicular pain and heaviness [4]. However, patients can rarely present with symptoms attributable to metastatic disease, and the involvement of the gastrointestinal (GI) tract with testicular germ cell tumors is exceedingly rare with seminomas being the least likely to metastasize to the GI tract [5]. Herein, we present the case of a 42-year-old male patient who presented with abdominal pain as the first main symptom of testicular seminoma with metastasis of the small bowel.

## Case presentation

A 42-year-old male without significant past medical or surgical history was admitted to a satellite hospital with complaints of progressive, intermittent right upper quadrant abdominal pain of one-month duration that increased in intensity and severity three days prior to his admission. The patient denied nausea, vomiting, heartburn, dysphagia, and overt GI bleeding. The patient also reported constipation and unintended weight loss of about 20lbs with loss of appetite over the past two months. 

**Figure 1:**
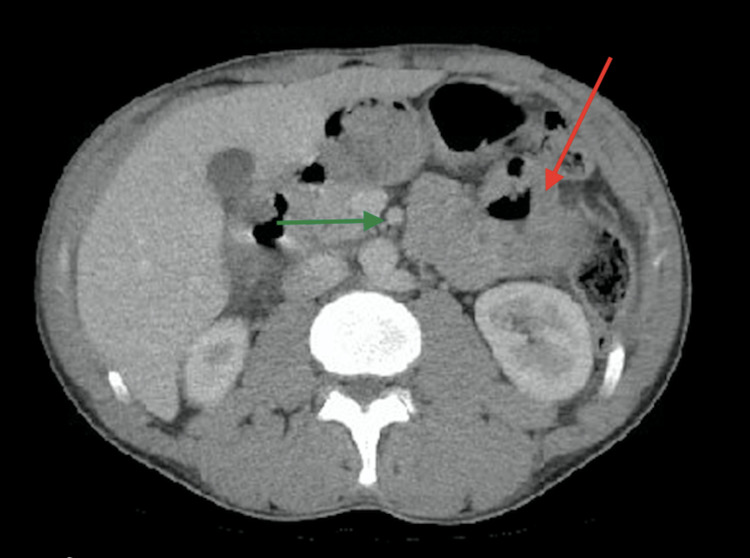
Transversal section of computed tomography scan of the abdomen Computed tomography scan demonstrating a mass-like thickening of the small bowel with dilated segments (red arrow) and scattered lymph nodes (green arrow).

Physical examination was only remarkable for a mildly distended abdomen with no rebound or guarding. The patient’s Initial laboratory results showed a white blood count of 8.2 K/mL, hemoglobin of 10.4 g/dl, and a hematocrit of 35.3%. His liver functions tests were normal. Computed tomography of the abdomen and pelvis with contrast on admission showed a mass-like thickening in the proximal small bowel with dilated segments and scattered enlarged proximal retroperitoneal and mesenteric lymph nodes (Figure 1).

**Figure 2:**
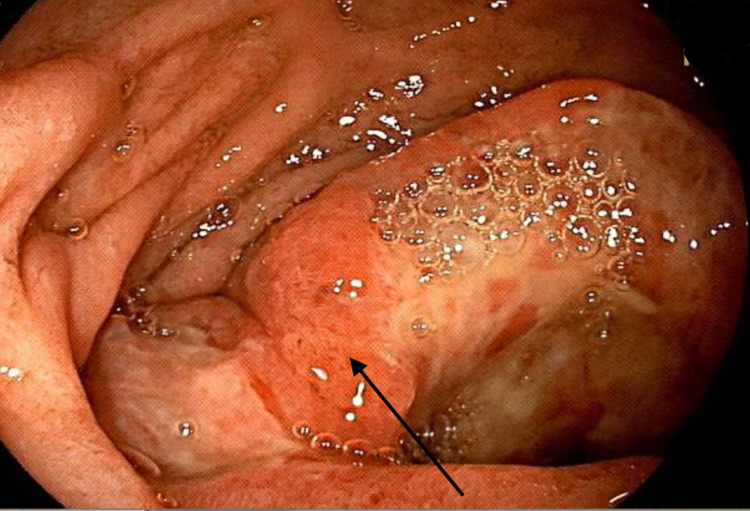
Endoscopic view of distal duodenum using push enteroscopy Endoscopic view revealing a friable, ulcerated, and fungating mass in the distal duodenum (black arrow).

The patient then underwent push enteroscopy revealing a mass-like lesion in the duodenum with lymph node involvement (Figure 2). Histopathology results revealed a malignant proliferation of large polygonal cells with a clear cytoplasm, in sheet-like arrangements. Immunostains showed expression of Sal‐like protein 4 (SALL4) and KIT (CD117) which are consistent with germ cell tumor. Although the patient denied any testicular pain, redness, or swelling and scrotal examination was unremarkable, scrotal ultrasound was performed and showed a hypoechoic right testicle with normal left testicle. Additional laboratory tests for serum tumor markers were performed and showed a lactic dehydrogenase level of 834 IU/L (normal levels between 105-300 IU/L), Human chorionic gonadotropin serum level of 3 mIU/mL (normal level less than 2 mIU/mL), alpha-fetoprotein level of 2.3 ng/mL (normal levels between 10-20 ng/mL), and a uric acid level of 5.5 mg/dL (normal levels between 3.4-7.0 mg/dL). The patient was diagnosed with metastatic testicular seminoma to the small bowel and was subsequently discharged home to be started on chemotherapy under the care of a multidisciplinary team led by the oncology department. He was also referred to urology for orchiectomy. 

## Discussion

We describe an unusual case of a 42-year-old man, who presented with abdominal pain as the initial manifestation of testicular seminoma with small bowel metastasis. Seminomas commonly metastasize to the lungs, bone, liver, and the retroperitoneal lymph nodes [1]. Testicular germ cell tumor metastasis to the upper GI tract occurs in less than 5% of non-seminomatous tumors, and in less than 1% of pure seminomas [5]. Metastasis to the GI tract usually occurs by direct extension through the retroperitoneal lymph nodes, and less commonly due to hematogenous spread or peritoneal seeding [6]. Testicular seminomas with GI metastasis have been reported in autopsies, and less frequently as a clinical disease [7].

The most common sites of metastasis involving the GI tract reported were the colon, esophagus, and stomach. Jejunal and iliac involvement were also found to be more common than duodenal involvement in these patients. The most frequently reported symptoms include diarrhea, nausea, vomiting, abdominal pain, and iron deficiency anemia [5,7]. One case reported a patient with testicular seminoma presenting with duodenal perforation [8]. These symptoms are usually related to either obstruction by the tumor, or bleeding and vascular invasion. Our patient presented with GI symptoms. In the majority of reported cases in literature, GI tract metastases in testicular seminomas were associated with natural progression of the disease and metastases in other sites. However, in our case, GI manifestations were not only the initial presentation, but also proximal small bowel involvement in the duodenum was an isolated metastasis, further contributing to the rarity of our findings [5,7]. Our case also highlights the continuing rise of the diagnostic value of endoscopy being relatively low risk and non-invasive. 

Although chemotherapy showed success in the treatment of metastatic seminoma, patients with GI metastasis are considered higher risk and generally are associated with poor outcomes [9]. The treatment in patients with metastasized testicular seminoma to the GI tract usually comprises of chemotherapy and/or surgical resection. The response rate to chemotherapy in such patients is approximately 60% [5,10].

## Conclusions

In conclusion, we report a unique case of small bowel metastasis as the initial presentation of testicular seminoma diagnosed by endoscopic tissue sampling. The metastasis to the small bowel in our patient was an isolated metastasis in the duodenum, which adds to the rarity of the presentation. Metastasis to the GI tract from the testis should be considered in the differential diagnosis of abdominal pain in young male patients, especially when associated with symptoms like unexplained weight loss, constitutional symptoms, and testicular pain or swelling. Furthermore, this case illustrates the importance of prompt diagnosis and treatment of such patients, as the delay in diagnosis can significantly impact the prognosis and survival in this patient population.
